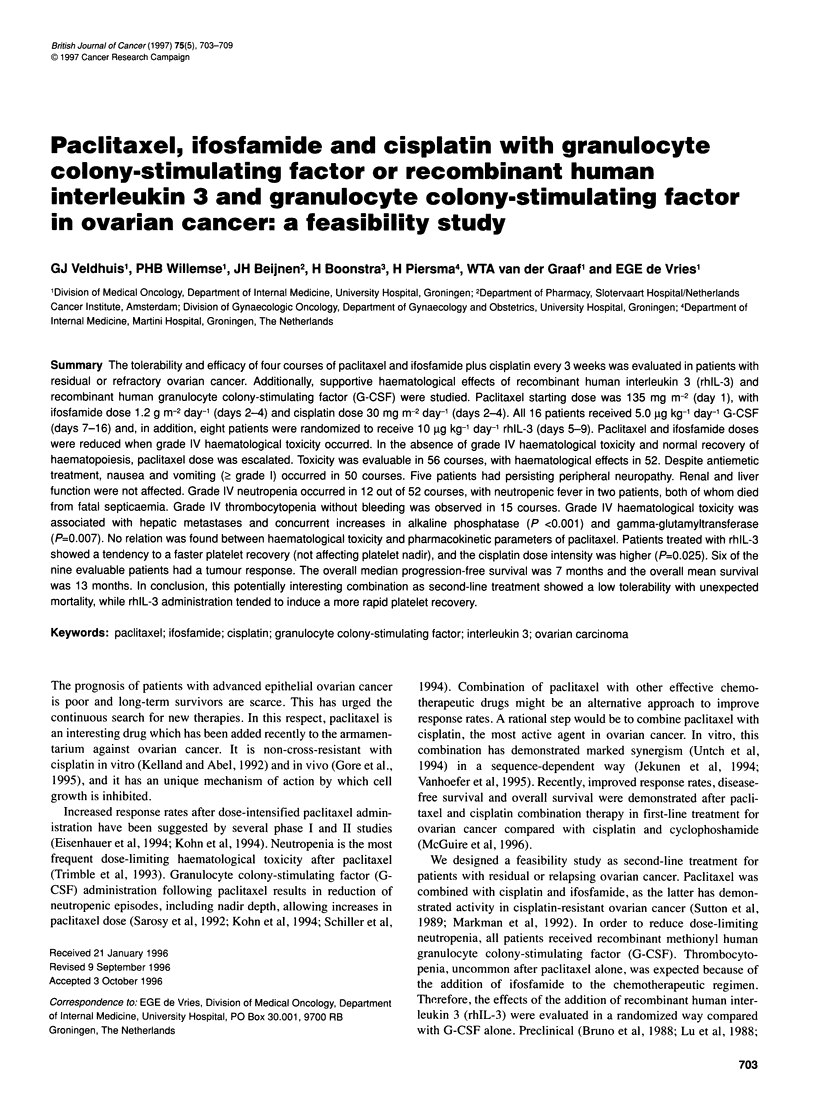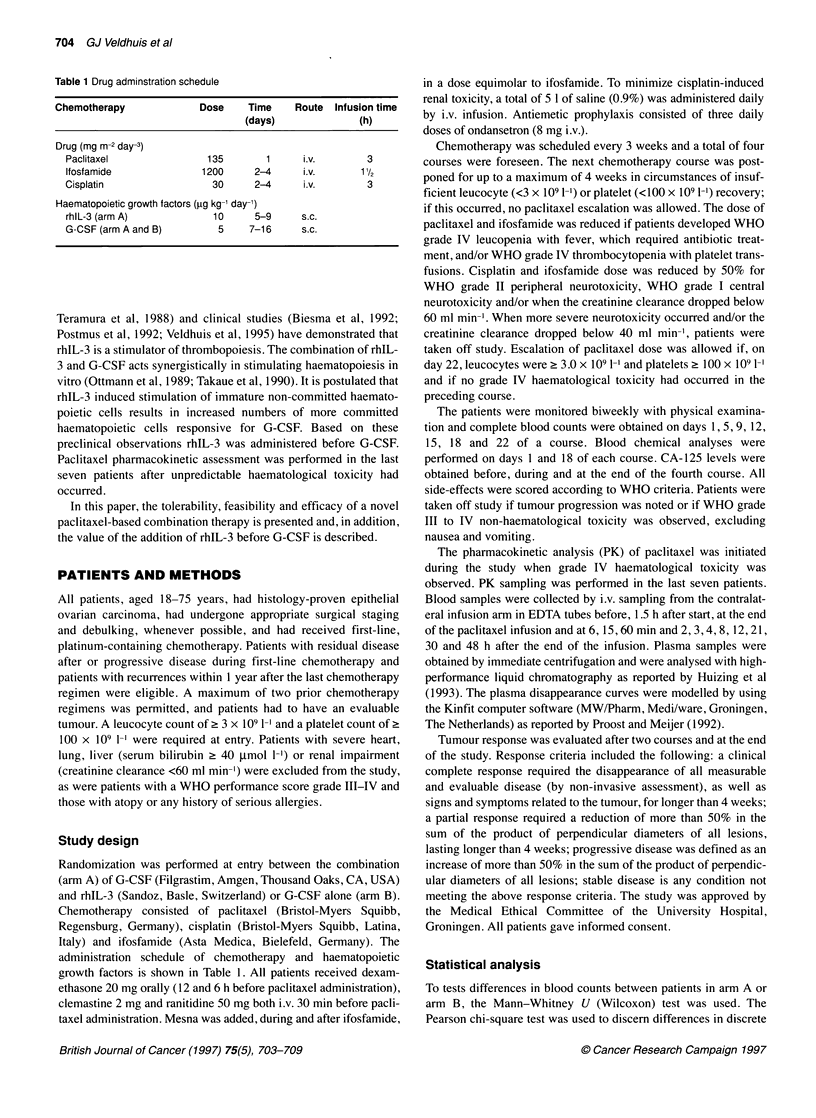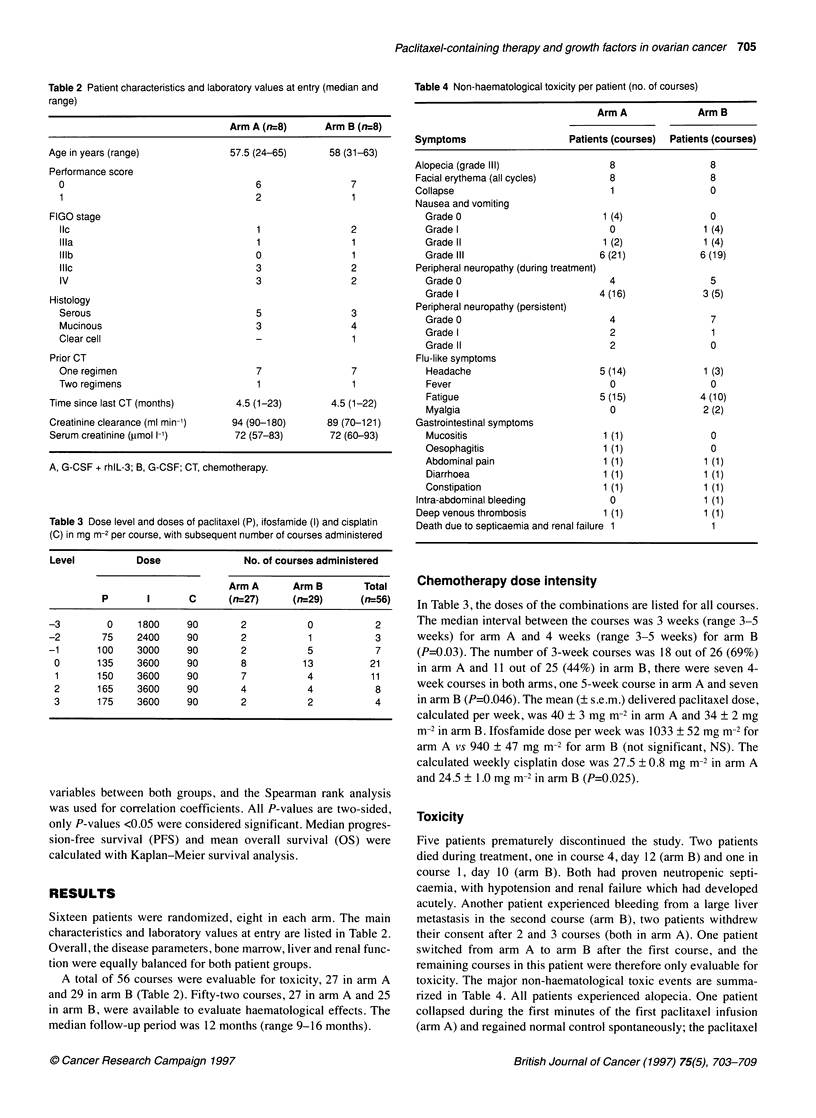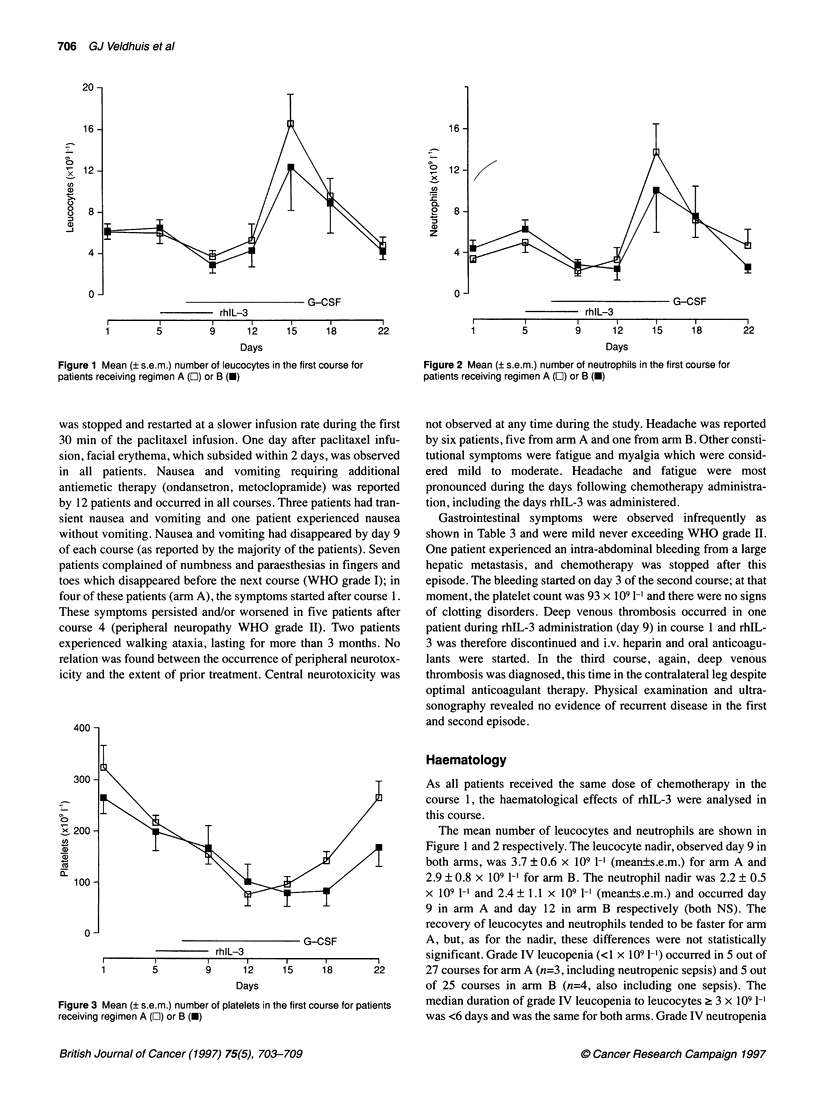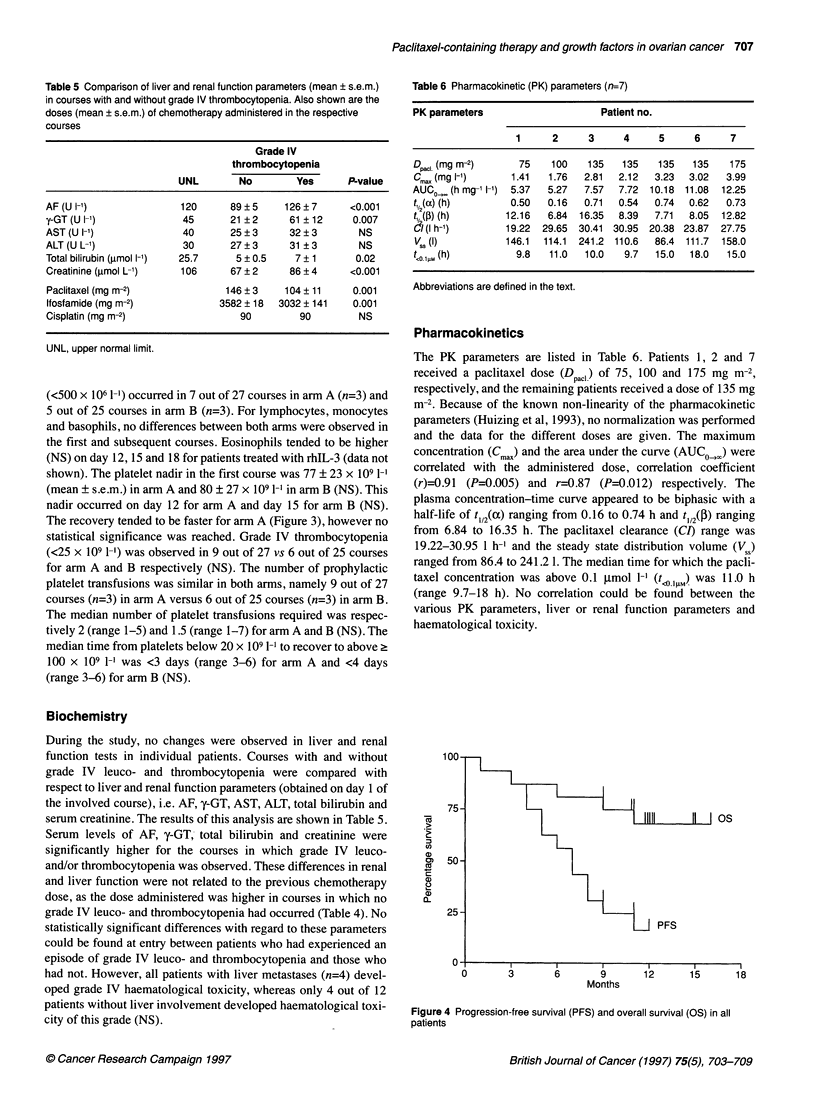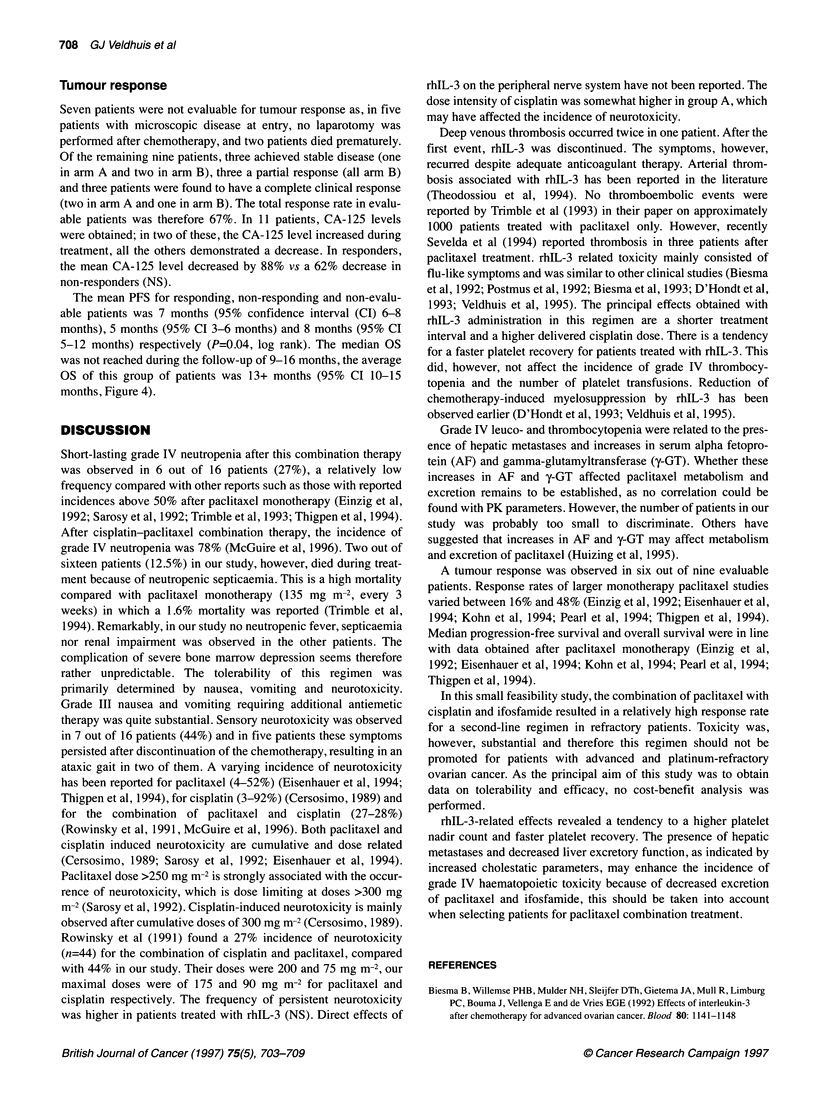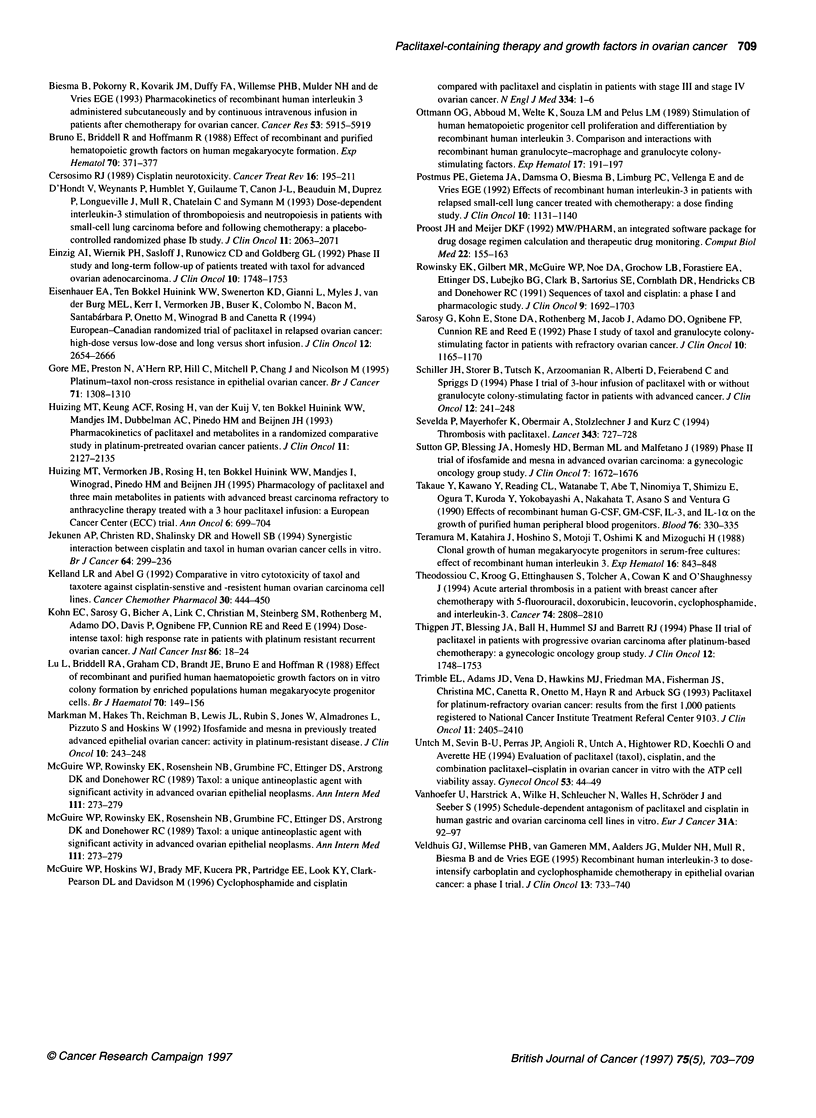# Paclitaxel, ifosfamide and cisplatin with granulocyte colony-stimulating factor or recombinant human interleukin 3 and granulocyte colony-stimulating factor in ovarian cancer: a feasibility study.

**DOI:** 10.1038/bjc.1997.125

**Published:** 1997

**Authors:** G. J. Veldhuis, P. H. Willemse, J. H. Beijnen, H. Boonstra, H. Piersma, W. T. van der Graaf, E. G. de Vries

**Affiliations:** Department of Internal Medicine, University Hospital, Groningen, The Netherlands.

## Abstract

The tolerability and efficacy of four courses of paclitaxel and ifosfamide plus cisplatin every 3 weeks was evaluated in patients with residual or refractory ovarian cancer. Additionally, supportive haematological effects of recombinant human interleukin 3 (rhIL-3) and recombinant human granulocyte colony-stimulating factor (G-CSF) were studied. Paclitaxel starting dose was 135 mg m(-2) (day 1), with ifosfamide dose 1.2 g m(-2) day(-1) (days 2-4) and cisplatin dose 30 mg m(-2) day(-1) (days 2-4). All 16 patients received 5.0 microg kg(-1) day(-1) G-CSF (days 7-16) and, in addition, eight patients were randomized to receive 10 microg kg(-1) day(-1) rhIL-3 (days 5-9). Paclitaxel and ifosfamide doses were reduced when grade IV haematological toxicity occurred. In the absence of grade IV haematological toxicity and normal recovery of haematopoiesis, paclitaxel dose was escalated. Toxicity was evaluable in 56 courses, with haematological effects in 52. Despite antiemetic treatment, nausea and vomiting (> or = grade I) occurred in 50 courses. Five patients had persisting peripheral neuropathy. Renal and liver function were not affected. Grade IV neutropenia occurred in 12 out of 52 courses, with neutropenic fever in two patients, both of whom died from fatal septicaemia. Grade IV thrombocytopenia without bleeding was observed in 15 courses. Grade IV haematological toxicity was associated with hepatic metastases and concurrent increases in alkaline phosphatase (P <0.001) and gamma-glutamyltransferase (P=0.007). No relation was found between haematological toxicity and pharmacokinetic parameters of paclitaxel. Patients treated with rhIL-3 showed a tendency to a faster platelet recovery (not affecting platelet nadir), and the cisplatin dose intensity was higher (P=0.025). Six of the nine evaluable patients had a tumour response. The overall median progression-free survival was 7 months and the overall mean survival was 13 months. In conclusion, this potentially interesting combination as second-line treatment showed a low tolerability with unexpected mortality, while rhIL-3 administration tended to induce a more rapid platelet recovery.


British Journal of Cancer (1997) 75(5), 703–709
© 1997 Cancer Research Campaign

### Paclitaxel, ifosfamide and cisplatin with granulocyte colony-stimulating factor or recombinant human interleukin 3 and granulocyte colony-stimulating factor in ovarian cancer: a feasibility study

**GJ Veldhuis[1], PHB Willemse[1], JH Beijnen[2], H Boonstra[3], H Piersma[4], WTA van der Graaf[1] and EGE de Vries[1]**

[1]Division of Medical Oncology, Department of Internal Medicine, University Hospital, Groningen; [2]Department of Pharmacy, Slotervaart Hospital/Netherlands Cancer Institute, Amsterdam; Division of Gynaecologic Oncology, Department of Gynaecology and Obstetrics, University Hospital, Groningen; [4]Department of Internal Medicine, Martini Hospital, Groningen, The Netherlands

**Summary** The tolerability and efficacy of four courses of paclitaxel and ifosfamide plus cisplatin every 3 weeks was evaluated in patients with residual or refractory ovarian cancer. Additionally, supportive haematological effects of recombinant human interleukin 3 (rhIL-3) and recombinant human granulocyte colony-stimulating factor (G-CSF) were studied. Paclitaxel starting dose was 135 mg $m^{-2}$ (day 1), with ifosfamide dose 1.2 g $m^{-2}$ $day^{-1}$ (days 2–4) and cisplatin dose 30 mg $m^{-2}$ $day^{-1}$ (days 2–4). All 16 patients received 5.0 μg $kg^{-1}$ $day^{-1}$ G-CSF (days 7–16) and, in addition, eight patients were randomized to receive 10 μg $kg^{-1}$ $day^{-1}$ rhIL-3 (days 5–9). Paclitaxel and ifosfamide doses were reduced when grade IV haematological toxicity occurred. In the absence of grade IV haematological toxicity and normal recovery of haematopoiesis, paclitaxel dose was escalated. Toxicity was evaluable in 56 courses, with haematological effects in 52. Despite antiemetic treatment, nausea and vomiting (≥ grade I) occurred in 50 courses. Five patients had persisting peripheral neuropathy. Renal and liver function were not affected. Grade IV neutropenia occurred in 12 out of 52 courses, with neutropenic fever in two patients, both of whom died from fatal septicaemia. Grade IV thrombocytopenia without bleeding was observed in 15 courses. Grade IV haematological toxicity was associated with hepatic metastases and concurrent increases in alkaline phosphatase ($P<0.001$) and gamma-glutamyltransferase ($P=0.007$). No relation was found between haematological toxicity and pharmacokinetic parameters of paclitaxel. Patients treated with rhIL-3 showed a tendency to a faster platelet recovery (not affecting platelet nadir), and the cisplatin dose intensity was higher ($P=0.025$). Six of the nine evaluable patients had a tumour response. The overall median progression-free survival was 7 months and the overall mean survival was 13 months. In conclusion, this potentially interesting combination as second-line treatment showed a low tolerability with unexpected mortality, while rhIL-3 administration tended to induce a more rapid platelet recovery.

**Keywords:** paclitaxel; ifosfamide; cisplatin; granulocyte colony-stimulating factor; interleukin 3; ovarian carcinoma

The prognosis of patients with advanced epithelial ovarian cancer is poor and long-term survivors are scarce. This has urged the continuous search for new therapies. In this respect, paclitaxel is an interesting drug which has been added recently to the armamentarium against ovarian cancer. It is non-cross-resistant with cisplatin in vitro (Kelland and Abel, 1992) and in vivo (Gore et al., 1995), and it has an unique mechanism of action by which cell growth is inhibited.

Increased response rates after dose-intensified paclitaxel administration have been suggested by several phase I and II studies (Eisenhauer et al, 1994; Kohn et al, 1994). Neutropenia is the most frequent dose-limiting haematological toxicity after paclitaxel (Trimble et al, 1993). Granulocyte colony-stimulating factor (G-CSF) administration following paclitaxel results in reduction of neutropenic episodes, including nadir depth, allowing increases in paclitaxel dose (Sarosy et al, 1992; Kohn et al, 1994; Schiller et al, 1994). Combination of paclitaxel with other effective chemotherapeutic drugs might be an alternative approach to improve response rates. A rational step would be to combine paclitaxel with cisplatin, the most active agent in ovarian cancer. In vitro, this combination has demonstrated marked synergism (Untch et al, 1994) in a sequence-dependent way (Jekunen et al, 1994; Vanhoefer et al, 1995). Recently, improved response rates, disease-free survival and overall survival were demonstrated after paclitaxel and cisplatin combination therapy in first-line treatment for ovarian cancer compared with cisplatin and cyclophoshamide (McGuire et al, 1996).

We designed a feasibility study as second-line treatment for patients with residual or relapsing ovarian cancer. Paclitaxel was combined with cisplatin and ifosfamide, as the latter has demonstrated activity in cisplatin-resistant ovarian cancer (Sutton et al, 1989; Markman et al, 1992). In order to reduce dose-limiting neutropenia, all patients received recombinant methionyl human granulocyte colony-stimulating factor (G-CSF). Thrombocytopenia, uncommon after paclitaxel alone, was expected because of the addition of ifosfamide to the chemotherapeutic regimen. Therefore, the effects of the addition of recombinant human interleukin 3 (rhIL-3) were evaluated in a randomized way compared with G-CSF alone. Preclinical (Bruno et al, 1988; Lu et al, 1988;

Received 21 January 1996
Revised 9 September 1996
Accepted 3 October 1996

*Correspondence to:* EGE de Vries, Division of Medical Oncology, Department of Internal Medicine, University Hospital, PO Box 30.001, 9700 RB Groningen, The Netherlands

Table 1 Drug adminstration schedule

| Chemotherapy | Dose | Time (days) | Route | Infusion time (h) |
|---|---|---|---|---|
| Drug (mg m$^{-2}$ day$^{-3}$) | | | | |
| Paclitaxel | 135 | 1 | i.v. | 3 |
| Ifosfamide | 1200 | 2–4 | i.v. | 1½ |
| Cisplatin | 30 | 2–4 | i.v. | 3 |
| Haematopoietic growth factors (μg kg$^{-1}$ day$^{-1}$) | | | | |
| rhIL-3 (arm A) | 10 | 5–9 | s.c. | |
| G-CSF (arm A and B) | 5 | 7–16 | s.c. | |

Teramura et al, 1988) and clinical studies (Biesma et al, 1992; Postmus et al, 1992; Veldhuis et al, 1995) have demonstrated that rhIL-3 is a stimulator of thrombopoiesis. The combination of rhIL-3 and G-CSF acts synergistically in stimulating haematopoiesis in vitro (Ottmann et al, 1989; Takaue et al, 1990). It is postulated that rhIL-3 induced stimulation of immature non-committed haematopoietic cells results in increased numbers of more committed haematopoietic cells responsive for G-CSF. Based on these preclinical observations rhIL-3 was administered before G-CSF. Paclitaxel pharmacokinetic assessment was performed in the last seven patients after unpredictable haematological toxicity had occurred.

In this paper, the tolerability, feasibility and efficacy of a novel paclitaxel-based combination therapy is presented and, in addition, the value of the addition of rhIL-3 before G-CSF is described.

### PATIENTS AND METHODS

All patients, aged 18–75 years, had histology-proven epithelial ovarian carcinoma, had undergone appropriate surgical staging and debulking, whenever possible, and had received first-line, platinum-containing chemotherapy. Patients with residual disease after or progressive disease during first-line chemotherapy and patients with recurrences within 1 year after the last chemotherapy regimen were eligible. A maximum of two prior chemotherapy regimens was permitted, and patients had to have an evaluable tumour. A leucocyte count of $\geq 3 \times 10^9$ l$^{-1}$ and a platelet count of $\geq 100 \times 10^9$ l$^{-1}$ were required at entry. Patients with severe heart, lung, liver (serum bilirubin $\geq$ 40 μmol l$^{-1}$) or renal impairment (creatinine clearance <60 ml min$^{-1}$) were excluded from the study, as were patients with a WHO performance score grade III–IV and those with atopy or any history of serious allergies.

### Study design

Randomization was performed at entry between the combination (arm A) of G-CSF (Filgrastim, Amgen, Thousand Oaks, CA, USA) and rhIL-3 (Sandoz, Basle, Switzerland) or G-CSF alone (arm B). Chemotherapy consisted of paclitaxel (Bristol-Myers Squibb, Regensburg, Germany), cisplatin (Bristol-Myers Squibb, Latina, Italy) and ifosfamide (Asta Medica, Bielefeld, Germany). The administration schedule of chemotherapy and haematopoietic growth factors is shown in Table 1. All patients received dexamethasone 20 mg orally (12 and 6 h before paclitaxel administration), clemastine 2 mg and ranitidine 50 mg both i.v. 30 min before paclitaxel administration. Mesna was added, during and after ifosfamide, in a dose equimolar to ifosfamide. To minimize cisplatin-induced renal toxicity, a total of 5 l of saline (0.9%) was administered daily by i.v. infusion. Antiemetic prophylaxis consisted of three daily doses of ondansetron (8 mg i.v.).

Chemotherapy was scheduled every 3 weeks and a total of four courses were foreseen. The next chemotherapy course was postponed for up to a maximum of 4 weeks in circumstances of insufficient leucocyte (<$3 \times 10^9$ l$^{-1}$) or platelet (<$100 \times 10^9$ l$^{-1}$) recovery; if this occurred, no paclitaxel escalation was allowed. The dose of paclitaxel and ifosfamide was reduced if patients developed WHO grade IV leucopenia with fever, which required antibiotic treatment, and/or WHO grade IV thrombocytopenia with platelet transfusions. Cisplatin and ifosfamide dose was reduced by 50% for WHO grade II peripheral neurotoxicity, WHO grade I central neurotoxicity and/or when the creatinine clearance dropped below 60 ml min$^{-1}$. When more severe neurotoxicity occurred and/or the creatinine clearance dropped below 40 ml min$^{-1}$, patients were taken off study. Escalation of paclitaxel dose was allowed if, on day 22, leucocytes were $\geq 3.0 \times 10^9$ l$^{-1}$ and platelets $\geq 100 \times 10^9$ l$^{-1}$ and if no grade IV haematological toxicity had occurred in the preceding course.

The patients were monitored biweekly with physical examination and complete blood counts were obtained on days 1, 5, 9, 12, 15, 18 and 22 of a course. Blood chemical analyses were performed on days 1 and 18 of each course. CA-125 levels were obtained before, during and at the end of the fourth course. All side-effects were scored according to WHO criteria. Patients were taken off study if tumour progression was noted or if WHO grade III to IV non-haematological toxicity was observed, excluding nausea and vomiting.

The pharmacokinetic analysis (PK) of paclitaxel was initiated during the study when grade IV haematological toxicity was observed. PK sampling was performed in the last seven patients. Blood samples were collected by i.v. sampling from the contralateral infusion arm in EDTA tubes before, 1.5 h after start, at the end of the paclitaxel infusion and at 6, 15, 60 min and 2, 3, 4, 8, 12, 21, 30 and 48 h after the end of the infusion. Plasma samples were obtained by immediate centrifugation and were analysed with high-performance liquid chromatography as reported by Huizing et al (1993). The plasma disappearance curves were modelled by using the Kinfit computer software (MW/Pharm, Medi/ware, Groningen, The Netherlands) as reported by Proost and Meijer (1992).

Tumour response was evaluated after two courses and at the end of the study. Response criteria included the following: a clinical complete response required the disappearance of all measurable and evaluable disease (by non-invasive assessment), as well as signs and symptoms related to the tumour, for longer than 4 weeks; a partial response required a reduction of more than 50% in the sum of the product of perpendicular diameters of all lesions, lasting longer than 4 weeks; progressive disease was defined as an increase of more than 50% in the sum of the product of perpendicular diameters of all lesions; stable disease is any condition not meeting the above response criteria. The study was approved by the Medical Ethical Committee of the University Hospital, Groningen. All patients gave informed consent.

### Statistical analysis

To tests differences in blood counts between patients in arm A or arm B, the Mann–Whitney *U* (Wilcoxon) test was used. The Pearson chi-square test was used to discern differences in discrete

 © Cancer Research Campaign 1997

**Table 2** Patient characteristics and laboratory values at entry (median and range)

| | Arm A (*n*=8) | Arm B (*n*=8) |
|---|---|---|
| Age in years (range) | 57.5 (24–65) | 58 (31–63) |
| Performance score | | |
| 0 | 6 | 7 |
| 1 | 2 | 1 |
| FIGO stage | | |
| IIc | 1 | 2 |
| IIIa | 1 | 1 |
| IIIb | 0 | 1 |
| IIIc | 3 | 2 |
| IV | 3 | 2 |
| Histology | | |
| Serous | 5 | 3 |
| Mucinous | 3 | 4 |
| Clear cell | – | 1 |
| Prior CT | | |
| One regimen | 7 | 7 |
| Two regimens | 1 | 1 |
| Time since last CT (months) | 4.5 (1–23) | 4.5 (1–22) |
| Creatinine clearance (ml min$^{-1}$) | 94 (90–180) | 89 (70–121) |
| Serum creatinine (μmol l$^{-1}$) | 72 (57–83) | 72 (60–93) |

A, G-CSF + rhIL-3; B, G-CSF; CT, chemotherapy.

**Table 3** Dose level and doses of paclitaxel (P), ifosfamide (I) and cisplatin (C) in mg m$^{-2}$ per course, with subsequent number of courses administered

| Level | Dose | | | No. of courses administered | | |
|---|---|---|---|---|---|---|
| | P | I | C | Arm A (*n*=27) | Arm B (*n*=29) | Total (*n*=56) |
| –3 | 0 | 1800 | 90 | 2 | 0 | 2 |
| –2 | 75 | 2400 | 90 | 2 | 1 | 3 |
| –1 | 100 | 3000 | 90 | 2 | 5 | 7 |
| 0 | 135 | 3600 | 90 | 8 | 13 | 21 |
| 1 | 150 | 3600 | 90 | 7 | 4 | 11 |
| 2 | 165 | 3600 | 90 | 4 | 4 | 8 |
| 3 | 175 | 3600 | 90 | 2 | 2 | 4 |

variables between both groups, and the Spearman rank analysis was used for correlation coefficients. All *P*-values are two-sided, only *P*-values <0.05 were considered significant. Median progression-free survival (PFS) and mean overall survival (OS) were calculated with Kaplan–Meier survival analysis.

### RESULTS

Sixteen patients were randomized, eight in each arm. The main characteristics and laboratory values at entry are listed in Table 2. Overall, the disease parameters, bone marrow, liver and renal function were equally balanced for both patient groups.

A total of 56 courses were evaluable for toxicity, 27 in arm A and 29 in arm B (Table 2). Fifty-two courses, 27 in arm A and 25 in arm B, were available to evaluate haematological effects. The median follow-up period was 12 months (range 9–16 months).

**Table 4** Non-haematological toxicity per patient (no. of courses)

| | Arm A | Arm B |
|---|---|---|
| Symptoms | Patients (courses) | Patients (courses) |
| Alopecia (grade III) | 8 | 8 |
| Facial erythema (all cycles) | 8 | 8 |
| Collapse | 1 | 0 |
| Nausea and vomiting | | |
| Grade 0 | 1 (4) | 0 |
| Grade I | 0 | 1 (4) |
| Grade II | 1 (2) | 1 (4) |
| Grade III | 6 (21) | 6 (19) |
| Peripheral neuropathy (during treatment) | | |
| Grade 0 | 4 | 5 |
| Grade I | 4 (16) | 3 (5) |
| Peripheral neuropathy (persistent) | | |
| Grade 0 | 4 | 7 |
| Grade I | 2 | 1 |
| Grade II | 2 | 0 |
| Flu-like symptoms | | |
| Headache | 5 (14) | 1 (3) |
| Fever | 0 | 0 |
| Fatigue | 5 (15) | 4 (10) |
| Myalgia | 0 | 2 (2) |
| Gastrointestinal symptoms | | |
| Mucositis | 1 (1) | 0 |
| Oesophagitis | 1 (1) | 0 |
| Abdominal pain | 1 (1) | 1 (1) |
| Diarrhoea | 1 (1) | 1 (1) |
| Constipation | 1 (1) | 1 (1) |
| Intra-abdominal bleeding | 0 | 1 (1) |
| Deep venous thrombosis | 1 (1) | 1 (1) |
| Death due to septicaemia and renal failure | 1 | 1 |

### Chemotherapy dose intensity

In Table 3, the doses of the combinations are listed for all courses. The median interval between the courses was 3 weeks (range 3–5 weeks) for arm A and 4 weeks (range 3–5 weeks) for arm B ($P$=0.03). The number of 3-week courses was 18 out of 26 (69%) in arm A and 11 out of 25 (44%) in arm B, there were seven 4-week courses in both arms, one 5-week course in arm A and seven in arm B ($P$=0.046). The mean (± s.e.m.) delivered paclitaxel dose, calculated per week, was 40 ± 3 mg m$^{-2}$ in arm A and 34 ± 2 mg m$^{-2}$ in arm B. Ifosfamide dose per week was 1033 ± 52 mg m$^{-2}$ for arm A *vs* 940 ± 47 mg m$^{-2}$ for arm B (not significant, NS). The calculated weekly cisplatin dose was 27.5 ± 0.8 mg m$^{-2}$ in arm A and 24.5 ± 1.0 mg m$^{-2}$ in arm B ($P$=0.025).

### Toxicity

Five patients prematurely discontinued the study. Two patients died during treatment, one in course 4, day 12 (arm B) and one in course 1, day 10 (arm B). Both had proven neutropenic septicaemia, with hypotension and renal failure which had developed acutely. Another patient experienced bleeding from a large liver metastasis in the second course (arm B), two patients withdrew their consent after 2 and 3 courses (both in arm A). One patient switched from arm A to arm B after the first course, and the remaining courses in this patient were therefore only evaluable for toxicity. The major non-haematological toxic events are summarized in Table 4. All patients experienced alopecia. One patient collapsed during the first minutes of the first paclitaxel infusion (arm A) and regained normal control spontaneously; the paclitaxel

© Cancer Research Campaign 1997

Figure 1 Mean (± s.e.m.) number of leucocytes in the first course for patients receiving regimen A (□) or B (■)

Figure 2 Mean (± s.e.m.) number of neutrophils in the first course for patients receiving regimen A (□) or B (■)

was stopped and restarted at a slower infusion rate during the first 30 min of the paclitaxel infusion. One day after paclitaxel infusion, facial erythema, which subsided within 2 days, was observed in all patients. Nausea and vomiting requiring additional antiemetic therapy (ondansetron, metoclopramide) was reported by 12 patients and occurred in all courses. Three patients had transient nausea and vomiting and one patient experienced nausea without vomiting. Nausea and vomiting had disappeared by day 9 of each course (as reported by the majority of the patients). Seven patients complained of numbness and paraesthesias in fingers and toes which disappeared before the next course (WHO grade I); in four of these patients (arm A), the symptoms started after course 1. These symptoms persisted and/or worsened in five patients after course 4 (peripheral neuropathy WHO grade II). Two patients experienced walking ataxia, lasting for more than 3 months. No relation was found between the occurrence of peripheral neurotoxicity and the extent of prior treatment. Central neurotoxicity was not observed at any time during the study. Headache was reported by six patients, five from arm A and one from arm B. Other constitutional symptoms were fatigue and myalgia which were considered mild to moderate. Headache and fatigue were most pronounced during the days following chemotherapy administration, including the days rhIL-3 was administered.

Gastrointestinal symptoms were observed infrequently as shown in Table 3 and were mild never exceeding WHO grade II. One patient experienced an intra-abdominal bleeding from a large hepatic metastasis, and chemotherapy was stopped after this episode. The bleeding started on day 3 of the second course; at that moment, the platelet count was 93 × $10^9$ $l^{-1}$ and there were no signs of clotting disorders. Deep venous thrombosis occurred in one patient during rhIL-3 administration (day 9) in course 1 and rhIL-3 was therefore discontinued and i.v. heparin and oral anticoagulants were started. In the third course, again, deep venous thrombosis was diagnosed, this time in the contralateral leg despite optimal anticoagulant therapy. Physical examination and ultrasonography revealed no evidence of recurrent disease in the first and second episode.

Figure 3 Mean (± s.e.m.) number of platelets in the first course for patients receiving regimen A (□) or B (■)

### Haematology

As all patients received the same dose of chemotherapy in the course 1, the haematological effects of rhIL-3 were analysed in this course.

The mean number of leucocytes and neutrophils are shown in Figure 1 and 2 respectively. The leucocyte nadir, observed day 9 in both arms, was 3.7 ± 0.6 × $10^9$ $l^{-1}$ (mean±s.e.m.) for arm A and 2.9 ± 0.8 × $10^9$ $l^{-1}$ for arm B. The neutrophil nadir was 2.2 ± 0.5 × $10^9$ $l^{-1}$ and 2.4 ± 1.1 × $10^9$ $l^{-1}$ (mean±s.e.m.) and occurred day 9 in arm A and day 12 in arm B respectively (both NS). The recovery of leucocytes and neutrophils tended to be faster for arm A, but, as for the nadir, these differences were not statistically significant. Grade IV leucopenia (<1 × $10^9$ $l^{-1}$) occurred in 5 out of 27 courses for arm A (*n*=3, including neutropenic sepsis) and 5 out of 25 courses in arm B (*n*=4, also including one sepsis). The median duration of grade IV leucopenia to leucocytes ≥ 3 × $10^9$ $l^{-1}$ was <6 days and was the same for both arms. Grade IV neutropenia

© Cancer Research Campaign 1997

**Table 5** Comparison of liver and renal function parameters (mean ± s.e.m.) in courses with and without grade IV thrombocytopenia. Also shown are the doses (mean ± s.e.m.) of chemotherapy administered in the respective courses

| | UNL | Grade IV thrombocytopenia No | Grade IV thrombocytopenia Yes | *P*-value |
|---|---|---|---|---|
| AF (U $l^{-1}$) | 120 | 89 ± 5 | 126 ± 7 | <0.001 |
| γ-GT (U $l^{-1}$) | 45 | 21 ± 2 | 61 ± 12 | 0.007 |
| AST (U $l^{-1}$) | 40 | 25 ± 3 | 32 ± 3 | NS |
| ALT (U $L^{-1}$) | 30 | 27 ± 3 | 31 ± 3 | NS |
| Total bilirubin (μmol $l^{-1}$) | 25.7 | 5 ± 0.5 | 7 ± 1 | 0.02 |
| Creatinine (μmol $L^{-1}$) | 106 | 67 ± 2 | 86 ± 4 | <0.001 |
| Paclitaxel (mg $m^{-2}$) | | 146 ± 3 | 104 ± 11 | 0.001 |
| Ifosfamide (mg $m^{-2}$) | | 3582 ± 18 | 3032 ± 141 | 0.001 |
| Cisplatin (mg $m^{-2}$) | | 90 | 90 | NS |

UNL, upper normal limit.

(<500 × $10^6$ $l^{-1}$) occurred in 7 out of 27 courses in arm A (*n*=3) and 5 out of 25 courses in arm B (*n*=3). For lymphocytes, monocytes and basophils, no differences between both arms were observed in the first and subsequent courses. Eosinophils tended to be higher (NS) on day 12, 15 and 18 for patients treated with rhIL-3 (data not shown). The platelet nadir in the first course was 77 ± 23 × $10^9$ $l^{-1}$ (mean ± s.e.m.) in arm A and 80 ± 27 × $10^9$ $l^{-1}$ in arm B (NS). This nadir occurred on day 12 for arm A and day 15 for arm B (NS). The recovery tended to be faster for arm A (Figure 3), however no statistical significance was reached. Grade IV thrombocytopenia (<25 × $10^9$ $l^{-1}$) was observed in 9 out of 27 *vs* 6 out of 25 courses for arm A and B respectively (NS). The number of prophylactic platelet transfusions was similar in both arms, namely 9 out of 27 courses (*n*=3) in arm A versus 6 out of 25 courses (*n*=3) in arm B. The median number of platelet transfusions required was respectively 2 (range 1–5) and 1.5 (range 1–7) for arm A and B (NS). The median time from platelets below 20 × $10^9$ $l^{-1}$ to recover to above ≥ 100 × $10^9$ $l^{-1}$ was <3 days (range 3–6) for arm A and <4 days (range 3–6) for arm B (NS).

### Biochemistry

During the study, no changes were observed in liver and renal function tests in individual patients. Courses with and without grade IV leuco- and thrombocytopenia were compared with respect to liver and renal function parameters (obtained on day 1 of the involved course), i.e. AF, γ-GT, AST, ALT, total bilirubin and serum creatinine. The results of this analysis are shown in Table 5. Serum levels of AF, γ-GT, total bilirubin and creatinine were significantly higher for the courses in which grade IV leuco- and/or thrombocytopenia was observed. These differences in renal and liver function were not related to the previous chemotherapy dose, as the dose administered was higher in courses in which no grade IV leuco- and thrombocytopenia had occurred (Table 4). No statistically significant differences with regard to these parameters could be found at entry between patients who had experienced an episode of grade IV leuco- and thrombocytopenia and those who had not. However, all patients with liver metastases (*n*=4) developed grade IV haematological toxicity, whereas only 4 out of 12 patients without liver involvement developed haematological toxicity of this grade (NS).

**Table 6** Pharmacokinetic (PK) parameters (*n*=7)

| PK parameters | Patient no. 1 | 2 | 3 | 4 | 5 | 6 | 7 |
|---|---|---|---|---|---|---|---|
| $D_{pacl.}$ (mg $m^{-2}$) | 75 | 100 | 135 | 135 | 135 | 135 | 175 |
| $C_{max}$ (mg $l^{-1}$) | 1.41 | 1.76 | 2.81 | 2.12 | 3.23 | 3.02 | 3.99 |
| $AUC_{0\to\infty}$ (h mg$^{-1}$ $l^{-1}$) | 5.37 | 5.27 | 7.57 | 7.72 | 10.18 | 11.08 | 12.25 |
| $t_{1/2}(\alpha)$ (h) | 0.50 | 0.16 | 0.71 | 0.54 | 0.74 | 0.62 | 0.73 |
| $t_{1/2}(\beta)$ (h) | 12.16 | 6.84 | 16.35 | 8.39 | 7.71 | 8.05 | 12.82 |
| *Cl* (l $h^{-1}$) | 19.22 | 29.65 | 30.41 | 30.95 | 20.38 | 23.87 | 27.75 |
| $V_{ss}$ (l) | 146.1 | 114.1 | 241.2 | 110.6 | 86.4 | 111.7 | 158.0 |
| $t_{<0.1\mu M}$ (h) | 9.8 | 11.0 | 10.0 | 9.7 | 15.0 | 18.0 | 15.0 |

Abbreviations are defined in the text.

### Pharmacokinetics

The PK parameters are listed in Table 6. Patients 1, 2 and 7 received a paclitaxel dose ($D_{pacl.}$) of 75, 100 and 175 mg $m^{-2}$, respectively, and the remaining patients received a dose of 135 mg $m^{-2}$. Because of the known non-linearity of the pharmacokinetic parameters (Huizing et al, 1993), no normalization was performed and the data for the different doses are given. The maximum concentration ($C_{max}$) and the area under the curve ($AUC_{0\to\infty}$) were correlated with the administered dose, correlation coefficient (*r*)=0.91 (*P*=0.005) and *r*=0.87 (*P*=0.012) respectively. The plasma concentration–time curve appeared to be biphasic with a half-life of $t_{1/2}(\alpha)$ ranging from 0.16 to 0.74 h and $t_{1/2}(\beta)$ ranging from 6.84 to 16.35 h. The paclitaxel clearance (*Cl*) range was 19.22–30.95 l $h^{-1}$ and the steady state distribution volume ($V_{ss}$) ranged from 86.4 to 241.2 l. The median time for which the paclitaxel concentration was above 0.1 μmol $l^{-1}$ ($t_{<0.1\mu M}$) was 11.0 h (range 9.7–18 h). No correlation could be found between the various PK parameters, liver or renal function parameters and haematological toxicity.

**Figure 4** Progression-free survival (PFS) and overall survival (OS) in all patients

### Tumour response

Seven patients were not evaluable for tumour response as, in five patients with microscopic disease at entry, no laparotomy was performed after chemotherapy, and two patients died prematurely. Of the remaining nine patients, three achieved stable disease (one in arm A and two in arm B), three a partial response (all arm B) and three patients were found to have a complete clinical response (two in arm A and one in arm B). The total response rate in evaluable patients was therefore 67%. In 11 patients, CA-125 levels were obtained; in two of these, the CA-125 level increased during treatment, all the others demonstrated a decrease. In responders, the mean CA-125 level decreased by 88% *vs* a 62% decrease in non-responders (NS).

The mean PFS for responding, non-responding and non-evaluable patients was 7 months (95% confidence interval (CI) 6–8 months), 5 months (95% CI 3–6 months) and 8 months (95% CI 5–12 months) respectively ($P$=0.04, log rank). The median OS was not reached during the follow-up of 9–16 months, the average OS of this group of patients was 13+ months (95% CI 10–15 months, Figure 4).

### DISCUSSION

Short-lasting grade IV neutropenia after this combination therapy was observed in 6 out of 16 patients (27%), a relatively low frequency compared with other reports such as those with reported incidences above 50% after paclitaxel monotherapy (Einzig et al, 1992; Sarosy et al, 1992; Trimble et al, 1993; Thigpen et al, 1994). After cisplatin–paclitaxel combination therapy, the incidence of grade IV neutropenia was 78% (McGuire et al, 1996). Two out of sixteen patients (12.5%) in our study, however, died during treatment because of neutropenic septicaemia. This is a high mortality compared with paclitaxel monotherapy (135 mg m$^{-2}$, every 3 weeks) in which a 1.6% mortality was reported (Trimble et al, 1994). Remarkably, in our study no neutropenic fever, septicaemia nor renal impairment was observed in the other patients. The complication of severe bone marrow depression seems therefore rather unpredictable. The tolerability of this regimen was primarily determined by nausea, vomiting and neurotoxicity. Grade III nausea and vomiting requiring additional antiemetic therapy was quite substantial. Sensory neurotoxicity was observed in 7 out of 16 patients (44%) and in five patients these symptoms persisted after discontinuation of the chemotherapy, resulting in an ataxic gait in two of them. A varying incidence of neurotoxicity has been reported for paclitaxel (4–52%) (Eisenhauer et al, 1994; Thigpen et al, 1994), for cisplatin (3–92%) (Cersosimo, 1989) and for the combination of paclitaxel and cisplatin (27–28%) (Rowinsky et al, 1991, McGuire et al, 1996). Both paclitaxel and cisplatin induced neurotoxicity are cumulative and dose related (Cersosimo, 1989; Sarosy et al, 1992; Eisenhauer et al, 1994). Paclitaxel dose >250 mg m$^{-2}$ is strongly associated with the occurrence of neurotoxicity, which is dose limiting at doses >300 mg m$^{-2}$ (Sarosy et al, 1992). Cisplatin-induced neurotoxicity is mainly observed after cumulative doses of 300 mg m$^{-2}$ (Cersosimo, 1989). Rowinsky et al (1991) found a 27% incidence of neurotoxicity ($n$=44) for the combination of cisplatin and paclitaxel, compared with 44% in our study. Their doses were 200 and 75 mg m$^{-2}$, our maximal doses were of 175 and 90 mg m$^{-2}$ for paclitaxel and cisplatin respectively. The frequency of persistent neurotoxicity was higher in patients treated with rhIL-3 (NS). Direct effects of rhIL-3 on the peripheral nerve system have not been reported. The dose intensity of cisplatin was somewhat higher in group A, which may have affected the incidence of neurotoxicity.

Deep venous thrombosis occurred twice in one patient. After the first event, rhIL-3 was discontinued. The symptoms, however, recurred despite adequate anticoagulant therapy. Arterial thrombosis associated with rhIL-3 has been reported in the literature (Theodossiou et al, 1994). No thromboembolic events were reported by Trimble et al (1993) in their paper on approximately 1000 patients treated with paclitaxel only. However, recently Sevelda et al (1994) reported thrombosis in three patients after paclitaxel treatment. rhIL-3 related toxicity mainly consisted of flu-like symptoms and was similar to other clinical studies (Biesma et al, 1992; Postmus et al, 1992; Biesma et al, 1993; D'Hondt et al, 1993; Veldhuis et al, 1995). The principal effects obtained with rhIL-3 administration in this regimen are a shorter treatment interval and a higher delivered cisplatin dose. There is a tendency for a faster platelet recovery for patients treated with rhIL-3. This did, however, not affect the incidence of grade IV thrombocytopenia and the number of platelet transfusions. Reduction of chemotherapy-induced myelosuppression by rhIL-3 has been observed earlier (D'Hondt et al, 1993; Veldhuis et al, 1995).

Grade IV leuco- and thrombocytopenia were related to the presence of hepatic metastases and increases in serum alpha fetoprotein (AF) and gamma-glutamyltransferase ($\gamma$-GT). Whether these increases in AF and $\gamma$-GT affected paclitaxel metabolism and excretion remains to be established, as no correlation could be found with PK parameters. However, the number of patients in our study was probably too small to discriminate. Others have suggested that increases in AF and $\gamma$-GT may affect metabolism and excretion of paclitaxel (Huizing et al, 1995).

A tumour response was observed in six out of nine evaluable patients. Response rates of larger monotherapy paclitaxel studies varied between 16% and 48% (Einzig et al, 1992; Eisenhauer et al, 1994; Kohn et al, 1994; Pearl et al, 1994; Thigpen et al, 1994). Median progression-free survival and overall survival were in line with data obtained after paclitaxel monotherapy (Einzig et al, 1992; Eisenhauer et al, 1994; Kohn et al, 1994; Pearl et al, 1994; Thigpen et al, 1994).

In this small feasibility study, the combination of paclitaxel with cisplatin and ifosfamide resulted in a relatively high response rate for a second-line regimen in refractory patients. Toxicity was, however, substantial and therefore this regimen should not be promoted for patients with advanced and platinum-refractory ovarian cancer. As the principal aim of this study was to obtain data on tolerability and efficacy, no cost-benefit analysis was performed.

rhIL-3-related effects revealed a tendency to a higher platelet nadir count and faster platelet recovery. The presence of hepatic metastases and decreased liver excretory function, as indicated by increased cholestatic parameters, may enhance the incidence of grade IV haematopoietic toxicity because of decreased excretion of paclitaxel and ifosfamide, this should be taken into account when selecting patients for paclitaxel combination treatment.

### REFERENCES

Biesma B, Willemse PHB, Mulder NH, Sleijfer DTh, Gietema JA, Mull R, Limburg PC, Bouma J, Vellenga E and de Vries EGE (1992) Effects of interleukin-3 after chemotherapy for advanced ovarian cancer. *Blood* **80**: 1141–1148

© Cancer Research Campaign 1997

Biesma B, Pokorny R, Kovarik JM, Duffy FA, Willemse PHB, Mulder NH and de Vries EGE (1993) Pharmacokinetics of recombinant human interleukin 3 administered subcutaneously and by continuous intravenous infusion in patients after chemotherapy for ovarian cancer. *Cancer Res* **53**: 5915–5919

Bruno E, Briddell R and Hoffmanm R (1988) Effect of recombinant and purified hematopoietic growth factors on human megakaryocyte formation. *Exp Hematol* **70**: 371–377

Cersosimo RJ (1989) Cisplatin neurotoxicity. *Cancer Treat Rev* **16**: 195–211

D'Hondt V, Weynants P, Humblet Y, Guilaume T, Canon J-L, Beauduin M, Duprez P, Longueville J, Mull R, Chatelain C and Symann M (1993) Dose-dependent interleukin-3 stimulation of thrombopoiesis and neutropoiesis in patients with small-cell lung carcinoma before and following chemotherapy: a placebo-controlled randomized phase Ib study. *J Clin Oncol* **11**: 2063–2071

Einzig AI, Wiernik PH, Sasloff J, Runowicz CD and Goldberg GL (1992) Phase II study and long-term follow-up of patients treated with taxol for advanced ovarian adenocarcinoma. *J Clin Oncol* **10**: 1748–1753

Eisenhauer EA, Ten Bokkel Huinink WW, Swenerton KD, Gianni L, Myles J, van der Burg MEL, Kerr I, Vermorken JB, Buser K, Colombo N, Bacon M, Santabárbara P, Onetto M, Winograd B and Canetta R (1994) European–Canadian randomized trial of paclitaxel in relapsed ovarian cancer: high-dose versus low-dose and long versus short infusion. *J Clin Oncol* **12**: 2654–2666

Gore ME, Preston N, A'Hern RP, Hill C, Mitchell P, Chang J and Nicolson M (1995) Platinum–taxol non-cross resistance in epithelial ovarian cancer. *Br J Cancer* **71**: 1308–1310

Huizing MT, Keung ACF, Rosing H, van der Kuij V, ten Bokkel Huinink WW, Mandjes IM, Dubbelman AC, Pinedo HM and Beijnen JH (1993) Pharmacokinetics of paclitaxel and metabolites in a randomized comparative study in platinum-pretreated ovarian cancer patients. *J Clin Oncol* **11**: 2127–2135

Huizing MT, Vermorken JB, Rosing H, ten Bokkel Huinink WW, Mandjes I, Winograd, Pinedo HM and Beijnen JH (1995) Pharmacology of paclitaxel and three main metabolites in patients with advanced breast carcinoma refractory to anthracycline therapy treated with a 3 hour paclitaxel infusion: a European Cancer Center (ECC) trial. *Ann Oncol* **6**: 699–704

Jekunen AP, Christen RD, Shalinsky DR and Howell SB (1994) Synergistic interaction between cisplatin and taxol in human ovarian cancer cells in vitro. *Br J Cancer* **64**: 299–236

Kelland LR and Abel G (1992) Comparative in vitro cytotoxicity of taxol and taxotere against cisplatin-senstive and -resistent human ovarian carcinoma cell lines. *Cancer Chemother Pharmacol* **30**: 444–450

Kohn EC, Sarosy G, Bicher A, Link C, Christian M, Steinberg SM, Rothenberg M, Adamo DO, Davis P, Ognibene FP, Cunnion RE and Reed E (1994) Dose-intense taxol: high response rate in patients with platinum resistant recurrent ovarian cancer. *J Natl Cancer Inst* **86**: 18–24

Lu L, Briddell RA, Graham CD, Brandt JE, Bruno E and Hoffman R (1988) Effect of recombinant and purified human haematopoietic growth factors on in vitro colony formation by enriched populations human megakaryocyte progenitor cells. *Br J Haematol* **70**: 149–156

Markman M, Hakes Th, Reichman B, Lewis JL, Rubin S, Jones W, Almadrones L, Pizzuto S and Hoskins W (1992) Ifosfamide and mesna in previously treated advanced epithelial ovarian cancer: activity in platinum-resistant disease. *J Clin Oncol* **10**: 243–248

McGuire WP, Rowinsky EK, Rosenshein NB, Grumbine FC, Ettinger DS, Arstrong DK and Donehower RC (1989) Taxol: a unique antineoplastic agent with significant activity in advanced ovarian epithelial neoplasms. *Ann Intern Med* **111**: 273–279

McGuire WP, Rowinsky EK, Rosenshein NB, Grumbine FC, Ettinger DS, Arstrong DK and Donehower RC (1989) Taxol: a unique antineoplastic agent with significant activity in advanced ovarian epithelial neoplasms. *Ann Intern Med* **111**: 273–279

McGuire WP, Hoskins WJ, Brady MF, Kucera PR, Partridge EE, Look KY, Clark-Pearson DL and Davidson M (1996) Cyclophosphamide and cisplatin compared with paclitaxel and cisplatin in patients with stage III and stage IV ovarian cancer. *N Engl J Med* **334**: 1–6

Ottmann OG, Abboud M, Welte K, Souza LM and Pelus LM (1989) Stimulation of human hematopoietic progenitor cell proliferation and differentiation by recombinant human interleukin 3. Comparison and interactions with recombinant human granulocyte–macrophage and granulocyte colony-stimulating factors. *Exp Hematol* **17**: 191–197

Postmus PE, Gietema JA, Damsma O, Biesma B, Limburg PC, Vellenga E and de Vries EGE (1992) Effects of recombinant human interleukin-3 in patients with relapsed small-cell lung cancer treated with chemotherapy: a dose finding study. *J Clin Oncol* **10**: 1131–1140

Proost JH and Meijer DKF (1992) MW/PHARM, an integrated software package for drug dosage regimen calculation and therapeutic drug monitoring. *Comput Biol Med* **22**: 155–163

Rowinsky EK, Gilbert MR, McGuire WP, Noe DA, Grochow LB, Forastiere EA, Ettinger DS, Lubejko BG, Clark B, Sartorius SE, Cornblath DR, Hendricks CB and Donehower RC (1991) Sequences of taxol and cisplatin: a phase I and pharmacologic study. *J Clin Oncol* **9**: 1692–1703

Sarosy G, Kohn E, Stone DA, Rothenberg M, Jacob J, Adamo DO, Ognibene FP, Cunnion RE and Reed E (1992) Phase I study of taxol and granulocyte colony-stimulating factor in patients with refractory ovarian cancer. *J Clin Oncol* **10**: 1165–1170

Schiller JH, Storer B, Tutsch K, Arzoomanian R, Alberti D, Feierabend C and Spriggs D (1994) Phase I trial of 3-hour infusion of paclitaxel with or without granulocyte colony-stimulating factor in patients with advanced cancer. *J Clin Oncol* **12**: 241–248

Sevelda P, Mayerhofer K, Obermair A, Stolzlechner J and Kurz C (1994) Thrombosis with paclitaxel. *Lancet* **343**: 727–728

Sutton GP, Blessing JA, Homesly HD, Berman ML and Malfetano J (1989) Phase II trial of ifosfamide and mesna in advanced ovarian carcinoma: a gynecologic oncology group study. *J Clin Oncol* **7**: 1672–1676

Takaue Y, Kawano Y, Reading CL, Watanabe T, Abe T, Ninomiya T, Shimizu E, Ogura T, Kuroda Y, Yokobayashi A, Nakahata T, Asano S and Ventura G (1990) Effects of recombinant human G-CSF, GM-CSF, IL-3, and IL-1$\alpha$ on the growth of purified human peripheral blood progenitors. *Blood* **76**: 330–335

Teramura M, Katahira J, Hoshino S, Motoji T, Oshimi K and Mizoguchi H (1988) Clonal growth of human megakaryocyte progenitors in serum-free cultures: effect of recombinant human interleukin 3. *Exp Hematol* **16**: 843–848

Theodossiou C, Kroog G, Ettinghausen S, Tolcher A, Cowan K and O'Shaughnessy J (1994) Acute arterial thrombosis in a patient with breast cancer after chemotherapy with 5-fluorouracil, doxorubicin, leucovorin, cyclophosphamide, and interleukin-3. *Cancer* **74**: 2808–2810

Thigpen JT, Blessing JA, Ball H, Hummel SJ and Barrett RJ (1994) Phase II trial of paclitaxel in patients with progressive ovarian carcinoma after platinum-based chemotherapy: a gynecologic oncology group study. *J Clin Oncol* **12**: 1748–1753

Trimble EL, Adams JD, Vena D, Hawkins MJ, Friedman MA, Fisherman JS, Christina MC, Canetta R, Onetto M, Hayn R and Arbuck SG (1993) Paclitaxel for platinum-refractory ovarian cancer: results from the first 1,000 patients registered to National Cancer Institute Treatment Referal Center 9103. *J Clin Oncol* **11**: 2405–2410

Untch M, Sevin B-U, Perras JP, Angioli R, Untch A, Hightower RD, Koechli O and Averette HE (1994) Evaluation of paclitaxel (taxol), cisplatin, and the combination paclitaxel–cisplatin in ovarian cancer in vitro with the ATP cell viability assay. *Gynecol Oncol* **53**: 44–49

Vanhoefer U, Harstrick A, Wilke H, Schleucher N, Walles H, Schröder J and Seeber S (1995) Schedule-dependent antagonism of paclitaxel and cisplatin in human gastric and ovarian carcinoma cell lines in vitro. *Eur J Cancer* **31A**: 92–97

Veldhuis GJ, Willemse PHB, van Gameren MM, Aalders JG, Mulder NH, Mull R, Biesma B and de Vries EGE (1995) Recombinant human interleukin-3 to dose-intensify carboplatin and cyclophosphamide chemotherapy in epithelial ovarian cancer: a phase I trial. *J Clin Oncol* **13**: 733–740

© Cancer Research Campaign 1997